# Inoculum Strategies and Performances of Malolactic Starter *Lactobacillus plantarum* M10: Impact on Chemical and Sensorial Characteristics of Fiano Wine

**DOI:** 10.3390/microorganisms8040516

**Published:** 2020-04-04

**Authors:** Silvia Jane Lombardi, Gianfranco Pannella, Massimo Iorizzo, Bruno Testa, Mariantonietta Succi, Patrizio Tremonte, Elena Sorrentino, Massimo Di Renzo, Daniela Strollo, Raffaele Coppola

**Affiliations:** 1 Department of Agricultural, Environmental and Food Sciences, University of Molise, 86100 Campobasso, Italy; silvia.lombardi@unimol.it (S.J.L.); gianfranco.pannella@unimol.it (G.P.); bruno.testa@studenti.unimol.it (B.T.); succi@unimol.it (M.S.); tremonte@unimol.it (P.T.); sorrentino@unimol.it (E.S.); coppola@unimol.it (R.C.); 2 Mastroberardino spa winery, via Manfredi, 75-81, 83042 Atripalda, Avellino, Italy; massimo.direnzo@mastroberardino.com (M.D.R.); daniela.strollo@mastroberardino.com (D.S.)

**Keywords:** *Lactobacillus plantarum*, malolactic fermentation, inoculum timing, Fiano wine

## Abstract

Malolactic fermentation (MLF) is a biological process that, in addition to deacidifying, also improves biological stability and changes the chemical and sensorial characteristics of wines. However, multiple biotic and abiotic factors, present in must and wine, make the onset and completion of MLF by indigenous malolactic bacteria or added commercial starters difficult. This work illustrates the metabolic and fermentative dynamics in winemaking Fiano wine, using a commercial starter of *Saccharomyces cerevisiae* and the selected strain *Lactobacillus plantarum* M10. In particular, an inoculum of malolactic starter was assessed at the beginning of alcoholic fermentation (early co-inoculum), at half alcoholic fermentation (late co-inoculum), and post alcoholic fermentation (sequential inoculum). The malolactic starter, before its use, was pre-adapted in sub-optimal growth conditions (pH 5.0). In sequential inoculum of the *Lb. plantarum* M10, even in a wine with high acidity, has confirmed its good technological and enzymatic characteristics, completing the MLF and enriching the wine with desirable volatile compounds.

## 1. Introduction

Malolactic fermentation (MLF) does not only represent the enzymatic conversion of L-malic acid to L-lactic acid and CO_2_, but it also includes other metabolic activities by lactic acid bacteria (LAB), which influence the aromatic compounds and provide biological stability to wine [1,2,3]. Generally, MLF takes place at the end of alcoholic fermentation and is mainly carried out by lactic acid bacteria belonging to two species: *Oenococcus oeni* and *Lb. plantarum.*

This fermentation may occur spontaneously or be induced by the addition of LAB commercial starters. Recently, their use has been preferred by the winemakers to guarantee more success in wine deacidification, also enhancing the sensorial features of wines. Scientific literature has highlighted that malolactic starters are selected according to very strict criteria and are able to survive in extremely difficult conditions [4,5,6,7,8].

However, despite current scientific and technical knowledge, the malolactic fermentation often remains unpredictable and difficult to manage. This is because the bacterial performances are strongly affected by different biological and chemical-physical variables: nutritional competition with yeasts, pH, medium-chain fatty acids, sulfur dioxide, alcohol levels. These problems stimulated the search for new and innovative strategies in the management of the winemaking process [9,10,11,12,13,14].

In recent years, apart from the search for new and increasingly effective starters, different inoculum techniques are used: sequential (after AF), co-inoculum (beginning or during alcoholic fermentation) and different forms of pre-adaptation of the malolactic starter [15,16,17]. Lately, a study has proved that *Lb. plantarum* strains long-term adapted to sub-optimal pH values better guarantee the course of the malolactic fermentation [17]. Moreover, several authors studying *Lb. plantarum* strains have described resistance mechanisms to high concentrations of ethanol and low pH, also evidencing that the stress response is variable and strain-dependent [18,19,20,21,22,23]. In addition, it has been demonstrated that *Lb. plantarum* strains during malolactic fermentation play an important role in wine quality definition [24,25]. In particular, their β-glycosidase activity release odorous compounds, including monoterpenes, that strongly influence the flavor profile of wine [26,27,28]. Despite that the influence of malolactic *Lb. plantarum* strains on the quality of wine has been widely investigated [29], our knowledge is still limited. Certain wine, such as Fiano, obtained from white grapes variety grown in Southern Italy, contain low levels of free volatile but are characterized by remarkably high concentrations of aromatic glycosylated precursors that can be transformed into odor-active volatiles during winemaking. In this respect, the β-glycosidase activity of *Lb. plantarum* can promote the release of odorous aglycones that increase the aromatic complexity of the wine [30,31,32,33,34]. So far, due to the hard-environmental condition characterizing Fiano wine, malolactic fermentation does not spontaneously take place and is difficult to manage even using commercial malolactic starters. 

Fiano grapes, despite being harvested later than other white grapes, are characterized by pH values of about 3.3, which are sub-lethal conditions for malolactic bacteria.

The present study monitors the evolution of the fermentation processes and the chemical profile of Fiano wines produced using a commercial *Saccharomyces cerevisiae* and selected strain *Lb. plantarum* M10.

In particular, we have investigated the effects of malolactic starter, pre-adapted in sub-optimal growth conditions, and used at the beginning, at half-time, or after alcoholic fermentation.

## 2. Materials and Methods

### 2.1. Strains, Media, Growth Conditions and Inoculum Preparation

In this work, the oenological commercial yeast *Saccharomyces cerevisiae* (Fermol Elegance, AEB, Brescia, Italy; indicated below with the abbreviation FE) and the bacterial strain *Lactobacillus plantarum* M10 were used. The M10 strain was taken up by a selection program aimed at selecting strains of *Lactobacillus plantarum* possessing good technological properties for malolactic fermentation, not a biogenic amine producer and possessing β-glucosidase activity [35]. The strain *S. cerevisiae* FE was rehydrated according to the manufacturer’s instructions, while the strain *Lb. plantarum* M10, stored at −80 °C in skim milk (Biolife, Milan, Italy), was propagated twice in de Man, Rogosa, and Sharpe (MRS) medium (Oxoid Ltd., Basingstoke, Hampshire, UK) at 28 °C before being used for inoculum preparation.

In order to obtain pre-adapted cells at sub-optimal pH, *Lb. plantarum* M10 was cultivated at 28 °C in MRS broth acidified at pH 5.0, using a laboratory-scale fermentation system (Biostat Aplus, Sartorius AG, Germany) with a working volume of 4.5 L, mixed at 200 rpm. Non-pre-adapted cells were obtained following the same procedure using MRS at pH 6.5 (Batch C). At the beginning of the stationary phase (approximately 10^9^ CFU/mL), the cells of both cultures were recovered and centrifuged at 10,000 rpm for 10 min at 4 °C. The supernatant was discarded and the pellet was washed twice with 1× of phosphate-buffered saline (PBS). Cells were suspended in filtrate must (with 0.4 µm membrane filter; ZP 130 Dalcin, Italy) and used to inoculate (10^7^ CFU/mL) [15].

### 2.2. Experimental Design of Winemaking Process

In this study, white grape of *Vitis vinifera cv* Fiano was used, harvested in the area of the city of Avellino (Italy) during the 2018 vintage. The winemaking process was carried out in a pilot plant in the Mastroberardino winery, located in the Campania region (Italy). The grape must show the following chemical composition: pH 3.24 ± 0.03, reducing sugars 216 g/L, titratable acidity 8.64 ± 0.12 (g/L tartaric acid), L-malic acid 3.44 g/L, L-lactic acid 0.04 g/L, acetic acid 0.01 g/L and YAN (yeast assimilable nitrogen) 158 ± 3 mg/L. To maintain an optimal YAN level for yeasts, after 72 h of fermentation, all the batches were added with 25 mg/L of ammonium phosphate. The wine fermentation process was performed according to the usual white wine winemaking. In detail, 50 mg/L of potassium metabisulphite was added to the grape must before fermentation. Then, five batches were set up and inoculated with about 10^7^ CFU/mL of *S. cerevisiae* FE. *Lb. plantarum* M10 (10^7^ CFU/mL) was added at different stages of the winemaking process, as detailed below.

Batch A: Grape must, was inoculated with *S. cerevisiae* FE and halfway through the alcoholic fermentation (about 5% ethanol) *Lb. plantarum* M10 was added (late co-inoculum); Batch B: *S. cerevisiae* FE and *Lb. plantarum* M10 were inoculated at the beginning of the alcoholic fermentation, after about 24 h, (early co-inoculum); Batch C: Grape must was inoculated with *S. cerevisiae* FE and at the end of the alcoholic fermentation *Lb. plantarum* M10 was added (sequential inoculum non pre-adapted); Batch D: Grape must was inoculated with *S. cerevisiae* FE and at the end of the alcoholic fermentation strain *Lb. plantarum* M10 was added (sequential inoculum pre-adapted); Batch E: Grape must was only inoculated with *S. cerevisiae* FE (control). In Batches C, D and E, at the end of the alcoholic fermentation, 20 g/L of nutrient for MLF (LATTittante, Dal Cin, Milan, Italy) was added, according to the usual practice in winery for malolactic fermentation. 

The experiments were performed in triplicates in stainless steel tanks (AISI 304 quality) of a working volume of 1 hL at 20 °C. The alcoholic (AF) and the malolactic (MLF) fermentation were monitored during the winemaking process at regular intervals. The alcoholic fermentation was considered finished when the reducing sugars were less than 2 g/L.

### 2.3. Microbiological Analyses

Colony counting was conducted in order to follow the growth trend of yeasts and lactic acid bacteria (LAB) during the winemaking processes. In detail, samples of different batches were picked up and at regular intervals (2 days), until the end of winemaking. One mL of sample was serially diluted with a sterile solution of peptone water (0.1%) and inoculated in appropriate media as follows: LAB were counted after 48 ± 2 h of incubation at 28 ± 1 °C in anaerobic conditions (GENbox anaer, bioMérieux, Marcy-l’Etoile, France) on MRS agar supplemented with 40 mg/L of cycloheximide; yeasts were detected on YPD agar (2% yeast extract, 2% *w/v* peptone and 4% *w/v* dextrose, 2% *w/v* agar), adding 30 mg/L of streptomycine, incubated at 28 ± 1 °C for 48 ± 2 h. 

### 2.4. Physico-Chemical Analyses

Physico-chemical analyses, performed on samples at the end of AF and on the 30th day of the winemaking process, were performed according to the corresponding European Community (EC) methods [36]. The malic acid and the lactic acid were determined using the enzymatic kit (Boehringer Mannheim, GmbH, Mannheim, Germany). Volatile compounds (mg/L) were determined by gas chromatography (GC) (Thermoquest Mod. 8000, Rodano, Milan, Italy) and flame ionization detection equipped with a fused capillary column ZB-Wax (30 m × 0.32 mm i.d., 0.50 μm film thickness, Phenomenex, Torrance CA, USA), according to International Organisation of Vine and Wine (OIV) methods [37]. The wines, after the addition of the internal standard (Butan-2-ol, Fluka, Steinheim, Germany; 0.1 mg/mL in water), were injected directly in split mode (1:50); injection port at 250 °C; program of oven from 40 °C (5min) to 240 °C at a rate of 7 °C/min; carrier gas helium with a flow rate of 60 kPa [29,38].

### 2.5. Sensory Analysis

In order to evaluate the different sensorial characteristics of the wines, at the end of the AF and MLF (Batch D), a sensorial analysis of the samples was performed by a panel of 12 professional testers from the National Organization of Wine Tasters (ONAV, Italy).

A tasting sheet was created in order to measure the intensity of each chosen descriptor using an unstructured intensity scale presented on a wheel. Three replicates of samples of each wine were sensory analyzed. The samples were presented randomly. This test was carried out in accordance with international standards. The sensory attributes assessed were for olfactory descriptors—fruity, herbal, floral, spicy, and for taste descriptors—reduced, acidity, persistence, astringency, softness. Finally, an overall judgment on the wine was expressed. Scores used ranged from 0 (absence of perception) to 9 (maximum perception) [39].

### 2.6. Statistical Analysis

Data obtained from three independent experiments were analyzed using the software RStudio (v 3.5.0) [40]. Analysis of variance (ANOVA) coupled with the Tukey HDS post-hoc test was used to evaluate differences between batches. Statistical significance was attributed to values of *p* < 0.05. Principal components analysis (PCA) was performed on wine volatile compounds to discriminate the volatiles between the different batches. 

## 3. Results and Discussion

### 3.1. Trend of Cell Biomass, Alcoholic and Malolactic Fermentation 

**Figure 1:**
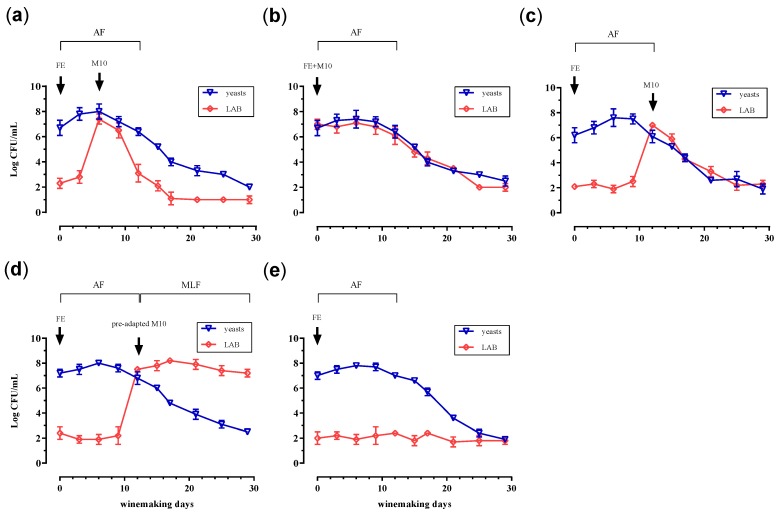
Trend of yeasts and LAB during the alcoholic (AF) and malolactic (MLF) fermentation of the 5 batches of Fiano grape must inoculated with *S. cerevisiae* FE at initial phase of the winemaking process and with *Lb. plantarum* M10 in accordance to different strategies of inoculum. (**a**) Batch inoculated with the strain M10 halfway through AF, late co-inoculum; (**b**) batch inoculated with M10 at the beginning of AF, early co-inoculum; (**c**) batch inoculated with *Lb. plantarum* M10 at the end of AF, sequential inoculum non-pre-adapted; (**d**) batch inoculated with M10 cells previously pre-adapted to acid and ethanol stress, sequential inoculum pre-adapted M10; (**e**) batch without inoculum of *Lb. plantarum* M10, control. The arrows indicate the point of M10 inoculum.

The evolution of the yeasts and LAB, as well as the trend of alcoholic and malolactic fermentation were followed during the winemaking process. In Figure 1, the development of the yeasts and the LAB during the alcoholic and malolactic fermentation is reported in the different batches. No differences in AF trend were noticed between the wines (Figure 1), whether they were inoculated with LAB (co-inoculum) or not (sequential inoculum). The results show that in all the batches, yeast cells increased rapidly after the inoculum, reaching the maximum density (≂8 Log CFU/mL) on the 6th day of fermentation. The cell concentration remained high ≂6 Log CFU/mL) until the 12th day, after which a progressive decay was observed until reaching a concentration of about 2 Log CFU/mL on the 29th day (end of winemaking). The quick beginning of the AF, observed in all trials, most likely indicates a good implantation of the yeasts.

**Figure 2:**
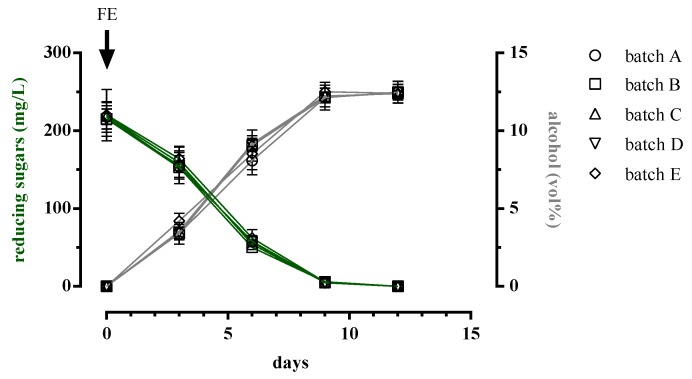
Consumption of reducing sugars and production of ethanol during the alcoholic fermentation of Fiano wine in accordance to several strategies of inoculum. Batch A, late co-inoculum strategy; Batch B, early co-inoculum strategy; Batch C, sequential inoculum non-pre-adapted strategy; Batch D, sequential inoculum pre-adapted strategy; Batch E, control.

The trend of yeast growth curves that we observed in all batches followed those of sugar consumption and alcohol production (Figure 2), highlighting that alcoholic fermentation was taking place. In fact, between the 9th and 12th day of the winemaking process, the reducing sugars were consumed and the alcohol content reached the maximal final value of about 12.5% (Figure 2) in all batches. 

These results highlight that the growth of the yeasts and AF were unaffected by the presence LAB. On the contrary, the authors reported the negative effect of LAB on yeast metabolism when used in co-inoculum [41,42,43], whilst other studies showed the positive effect on the growth and fermentative activity of yeast when it was co-inoculated with the LAB in grape must [44,45,46]. Therefore, the choice of the yeast and bacterial strains is crucial to avoid the onset of negative interactions between them. 

**Figure 3:**
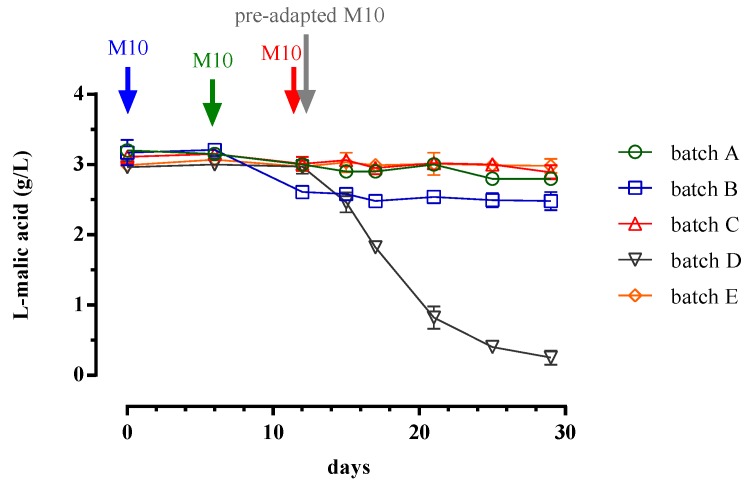
Consumption of L-malic acid during the Fiano winemaking process after the inoculum of *Lb. plantarum* M10 according to several strategies. Batch A, late co-inoculum strategy; Batch B, early co-inoculum strategy; Batch C, sequential inoculum non-pre-adapted strategy; Batch D, sequential inoculum pre-adapted strategy; Batch E, control. The arrows indicate the time of *Lb. plantarum* M10 inoculum.

Relative to LAB trend, several differences were observed between batches in response to the inoculation strategy. As reported in Figure 1, the survival of the bacteria was affected by the time of inoculum as well as the pre-adaption to sub-optimal pH condition. We observed a strong decay of bacteria survival, when non-preadapted *Lb. plantarum* M10 was inoculated according to sequential inoculum strategy (Figure 1c). In fact, at about 16 days (from days 12 to 16) of the winemaking process, the LAB load decreased from 7 to about 4 Log CFU/mL. The reduction of bacteria, when inoculated in the wine at the end of AF, was also evidenced by the failure of MLF. In fact, as evidenced in Figure 3, no difference was observed, in terms of L-malic acid consumption, following the inoculum of *Lb. plantarum* M10 (Batch C) in the wine. The loss of survival of malolactic starter and consequently the failure of MLF, is a negative event that frequently occurs during the winemaking process because of harsh conditions of wine, such as low pH, high ethanol contents, and low sugar concentration [42,47].

The worst-case scenario (Figure 1a) was observed when *Lb. plantarum* M10 was added to the grape must halfway through alcoholic fermentation (late co-inoculum). In this case, the microbial load was reduced to about 5 Log CFU/mL in the six days following the inoculation (from days 6 to 12), then reached values near the detection limit (1 Log CFU/mL). Consequently, as shown in Figure 3, the malolactic fermentation was also compromised. It is probable that the prohibitive conditions of the environment, associated with a potential competitive action of the yeast for nutrients, could lead to a major reduction of LAB concentration in late co-inoculum strategy, compared to the sequential inoculum approach. In fact, when the strain M10 was inoculated in the must, according to the late co-inoculum strategy (Figure 1a), it encountered more competitive cells of yeast because they were in the stationary phase. On the contrary, in the sequential inoculum, the yeast cells were in the decline phase at the moment of M10 inoculum and were, therefore, less competitive (Figure 1c). Similar results were carried out in a work conducted by Rosi et al. [48], where the authors reported the antagonist effect of yeast against *Oenococcus oeni*. 

Regarding the early co-inoculum method (Figure 1b), the reduction of LAB concentration was also recorded during the winemaking period. However, in this case, the survival curve of bacteria was less pronounced compared to those observed in the late co-inoculum and in the sequential inoculum non-preadapted (Figure 1a,c). In detail, after the inoculum *Lb. plantarum* M10 (7 Log CFU/mL), the number of viable cells remained constant until the 9th day of AF; subsequently, it decreased up to about 2 Log CFU/mL (30th day). In this case, a decrease in the L-malic acid concentration (about 0.5 g/L) was also observed (Figure 3, Batch B). In particular, the degradation of L-malic acid was triggered on the 6th day of AF, when the concentration of LAB was of about 7 Log CFU/mL. Following the reduction of bacterial load, under 6 Log CFU/mL (after the 12th day), the concentration of malic acid remained unchanged. These results once again highlight that the L-malic acid degradation is strongly correlated to an appropriate concentration of malolactic starter culture. 

This hypothesis was confirmed by the results obtained when *Lb. plantarum* M10 was pre-adapted to sub-optimal pH and subsequently inoculated in the wine at the end of alcoholic fermentation (Figure 1d). In this Batch (D), the microbial load (7.5 Log CFU/mL) was unchanged until the end of winemaking process (30th day), and consequently the MLF was also carried out (Figure 3). 

In detail, from the 12th day (inoculum of *Lb. plantarum* M10) to the 30th day (end of winemaking), the L-malic acid was completely degraded, going from values of about 3 to 0.2 g/L. Moreover, with the degradation of malic acid, there was a significant increase in lactic acid and pH, as can be seen from the physico-chemical parameters reported in Table 1. 

This finding shows how the pre-adaptation of LAB strain can positively influence both survival and L-malic acid degradation in the wine. The positive impact of cell pre-adaption to acid stress on microbial survival has also been reported in several studies [6,49,50]. Moreover, the same authors reported that the cells of *Lb. plantarum* pre-adapted to acid stress (pH 5.0); not only were they able to survive better in wine-like medium but showed a greater ability to degrade malic acid compared to non-pre-adapted cells [16].

The success of sequential inoculation compared to co-inoculation may also be due to other factors. In co-inoculation, the antagonistic effect attributed to yeast, linked to nutritional competition or the presence of medium chain fatty acids, can compromise the vitality of malolactic bacteria. On the contrary, in sequential inoculation, the yeast can favor the growth of LAB, releasing vitamins and amino acids after its autolysis. This involves enrichment of nutrients and subsequent stimulation of MLF.

### 3.2. Wine Chemical Analysis

The analyses of chemical parameters carried out in the wines obtained from Batches A, B, C, and E did not show significant differences after the AF and after 30 days of the winemaking process (data not shown). However, in Batch D after the MLF, the chemical compositions were significantly different from those observed in the other batches. In particular, as highlighted in Table 1, an increase in pH values (from 3.25 to 3.68) and in L-lactic acid levels (from 0.13 to 2.23) and a decrease in total acidity (from 8.54 to 6.52) as well as in L-malic acid (from 3.18 to 0.25) were registered. 

A total of 19 volatile compounds were identified in the Fiano wine at the end of the winemaking process (30th day) and their concentration is reported in Table 2. In particular, two main classes of compounds were detected, distinguishable in higher alcohols and terpenes. These compounds, together, contributed to the complexity of the wine flavor. 

Higher alcohols represent the major volatiles detected in the samples. Fourteen compounds were identified, and their concentration ranged from 984.18 to 1071.59 mg/L, respectively, in the wines from Batches C and B. Among all identified alcohol, 2,3-butanediol, 3-methyl-1-butanol, and 1-heptanol were the most abundant in all wines, contributing up to 77% of the whole concentration. 

2,3-butanediol is a compound related to a fruity note of wine [51,52,53,54]; it is obtained from the reduction of diacetyl (2,3-butanedione) by means of diacetyl reductase. 

2,3-butanedione can be produced during alcohol fermentation as an intermediate product of amino acids metabolism of yeast, and through malolactic fermentation in acid citric metabolism. Consequently, a major content of 2,3-butanediol could be detected after MLF [51,52,53,55]. In our study, the concentration of 2,3-butanediol (Table 2) was significantly higher in wine where MLF occurred (Batch D) compared to wines where MLF failed (Batches A and B).

3-methyl-1-butanol (iso-amyl alcohol) was detected in high concentrations in all wines, even if relevant differences were observed among the batches. In particular, we observed a lower significant concentration of 3-methyl-1-butanol in wine where MLF occurred (Batch D), and the highest concentration in the wines where the yeast and bacteria coexisted (Batches A and B). Although, 3-methyl-1-butanol is a metabolite produced by yeast, through the metabolism of amino acids leucine and valine, or by metabolism of pyruvate [56], our results highlight that concentration of iso-amyl alcohol is strongly influenced by yeast-bacteria interaction, according to results reported by Lee et al. [21]. 3-methyl-1-butanol gives a banana and pear-like aroma at optimal concentration, but excessive concentrations are responsible of the nail polish odor of wines [56,57,58].

The concentrations of 1-octanol were above their aroma detection thresholds in all samples, but more in Batches A and B, indicating a direct impact on the aroma characteristics of these wines. Some authors believe that 2-phenylethanol does not derive de novo from yeasts but originates from the aglycosidically-bound form in the fruit, which is liberated during fermentation [59,60,61] as a result of enzyme activity. In fact, higher concentration of 2-phenylethanol was observed in Batch D, while the concentration significantly decreased in wines where the MLF was absent (Batches A, B, C, and E), which proved an enzymatic activity was carried out by the malolactic bacteria.

In this study, the concentration of some higher alcohols, 1-propanol, 1-methyl-propanol, 2-pentanol, and methyl-tyo-propanol, significantly decreased. Maicas et al. [55] reported the important influence of the LAB strain participating in MLF on the evolution of alcohol concentrations, and affirmed that the decreases observed when certain strains were used may have been due to the physical adsorption of the bacteria.

Linalool, 4-terpineol, α-terpineol, and geraniol were the esters quantified in wines from different batches. Wine from Batch D, obtained with pre-adapted inoculum of *Lb. plantarum* M10, showed a significantly higher concentration in linalool (124.50 µg/L) compared to the other batches (Table 2); the levels of 4-terpineol were also higher (18.07 µg/L), which are expression of the varietal aroma characteristics of Fiano wine [33]. Furthermore, terpenes are known to bring desirable floral and citrus aroma, a contribution of this compound to the aroma of young white wines.

Higher concentration of hexanoic acid in Batches A and B, where the co-inoculum was carried out, could be one of the causes of the inhibition malolactic fermentation.

Some researchers reported that short- to medium-chain fatty acids, such as hexanoic, octanoic and decanoic acids, produced during alcoholic fermentation, inhibit the growth of malolactic bacteria [62,63,64]. Production of these acids varies significantly with yeast species and strain. Though, the nature of the antagonism between yeast and malolactic bacteria remains unclear.

### 3.3. Principal Component Analysis

**Figure 4:**
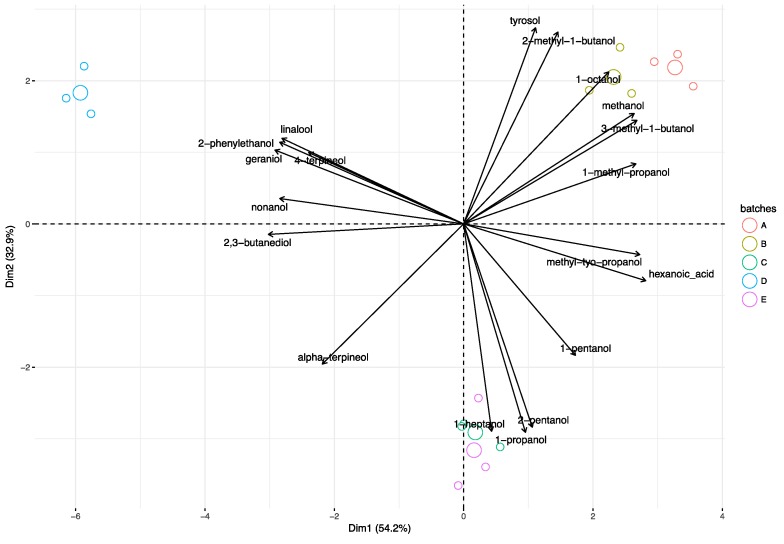
Principal components analysis (PCA)-biplot of volatile compounds in different batches at the end of the winemaking process. The big circles represent the mean values of three independent experiments.

The principal component analysis was applied to volatile compounds detected in the wines of five batches at the end of the winemaking process (30 days). The first two principal components (Dim 1 and Dim 2) displayed about 87% of variance, of which 54% was due to Component 1 (Figure 4). The volatile compounds profile of wines was clearly distinguishable along the coordinates of the two principal components. Batch A and Batch B were divergent from Batch D across the Dim 1; moreover they were also separated from Batches C and E across the Dim 2 (Figure 4). From the bi-plot, it is evident that the presence of *Lb. plantarum* M10 as well as the timing of inoculations in the winemaking process, could modulate the volatile compounds of wines. Wines located on the upper right-hand side of the bi-plot (Figure 4), obtained by the early inoculum (Batch A) and co-inoculum (Batch B) of strains FE and M10, were mainly characterized by 1-octanol, methanol, and 3-methyl-1-butanol. The sample wines, placed on the lower right quadrant of bi-plot (Figure 4) and achieved by the sequential inoculum (Batch C) of strains FE and M10 or without the inoculum of M10 (Batch E), showed aromatic profiles mainly influenced by the compounds 1-heptanol, 1-propanol, and 2-pentanol, whilst the wine (Batch D) obtained with the pre-adapted sequential inoculum strategy, located on the upper left-hand side of the two principal components, showed an aromatic profile attributable to linalool, 2-phenylethanol, 4-terpineol, geraniol, and 2,3-butanediol.

### 3.4. Sensory Evaluation

**Figure 5:**
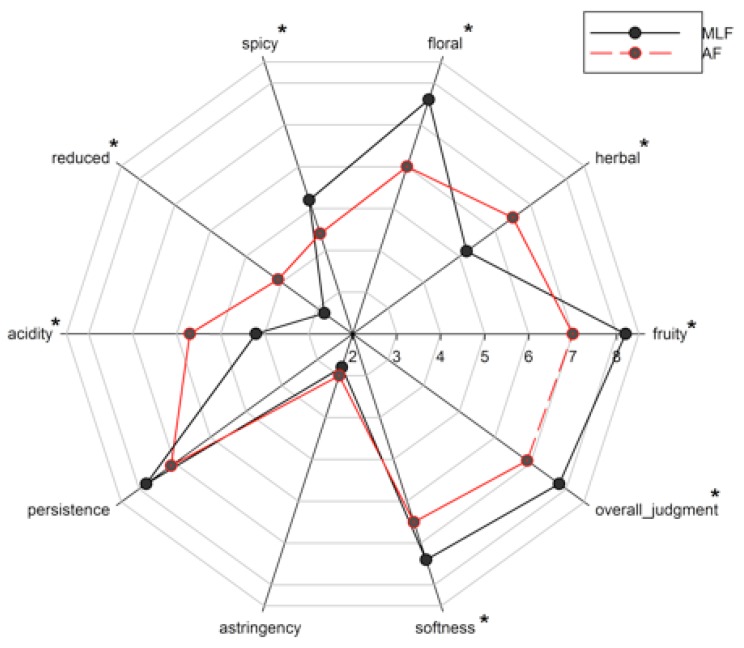
Radar plot of Fiano wine obtained by sequential inoculum strategy with M10 cells pre-adapted to acid stress. The two samples evaluated come from Batch D, at the end of alcoholic fermentation (AF) and at the end of malolactic fermentation (MLF). * indicates significant differences (*p* < 0.05) of attributes between AF and MLF.

In order to confirm the results obtained with the analysis of volatile compounds, the sensorial analysis was performed only on the sample where malic acid was consumed (Batch D), evaluating the differences of the wine at the end of the alcoholic fermentation (AF) and at the malolactic fermentation performed (MLF). The judges found that the wine at the end of MLF had best overall quality and gave a score around 8 points (Figure 5). Furthermore, comparing sensory variation between wines (AF, MLF), it varied significantly in all attributes except for the astringency and persistence. The aroma of the wine MLF was mainly characterized by fruit and floral notes, while the attribute “reduced” was especially low. The increase in the concentration of some volatile compounds seems to show the determining contribution of the β-glucosidasic activity of lactic acid bacteria to wine flavor [33]. The sensations perceived by the sensorial analysis of the analyzed samples are related to the greater quantity of odorous compounds chemically determined.

## 4. Conclusions

The improvement of the quality of the wine is linked not only to the quality of the raw material, but also to the winemaking techniques and the starters used.

Malolactic fermentation (MLF) is “secondary” in wine only chronologically but, like alcoholic fermentation (AF), a “primary” process by importance in defining the quality of wine.

In this study, the success of *Lb. plantarum* M10, suitably pre-adapted and inoculated after alcoholic fermentation and used as a malolactic starter, testifies the importance of the inoculation technique and the characteristics of the strain used. Furthermore, metabolic activities are strongly linked to the ability to survive specific stress factors found in wine (mainly high ethanol content and low pH). Stress responses have been correlated with specific phenotypes so that they can be induced in a controllable and reproducible way. Furthermore, the use of sequential inoculation was more favorable, probably due to the lack of adverse interaction between yeast and bacterium. These results indicate that, despite the very low pH of the wine, correct management of the inoculation strategies can determine both the success of the malolactic fermentation and the improvement of the sensorial characteristics of the wines. In this case, the sequential inoculum combined with the pre-adaptation at sub-optimal pH proved to be decisive.

## Figures and Tables

**Table 1:** Physico-chemical analysis of wine at the end of alcoholic fermentation (AF) and at the end of malolactic fermentation (MLF). The wine was obtained with the sequential inoculation pre-adapted (Batch D).

Physico-Chemical Parameters	AF			MLF		
pH	3.25	±	0.08 *	3.68	±	0.09
Total acidity (g/L)	8.54	±	0.16 *	6.52	±	0.23
Reducing sugars (g/L)	1.10	±	0.05 *	0.75	±	0.17
Alcohol (% vol)	12.58	±	0.17	12.55	±	0.13
Volatile acidity (g/L)	0.31	±	0.07	0.42	±	0.04
L-malic acid (g/L)	3.18	±	0.06 *	0.25	±	0.11
L-lactic acid (g/L)	0.13	±	0.03 *	2.23	±	0.07
Free SO_2_ (mg/L)	11.21	±	0.41	11.34	±	0.44
Total SO_2_ (mg/L)	45.16	±	1.13	48.23	±	1.75

Mean values of three independent replicate ± standard deviation. * indicates significant differences (*p* < 0.05) between values obtained in wine after AF and wine at the end of MLF.

**Table 2:** Concentration of volatile compounds at the end of winemaking process of Fiano wines produced by different inoculum strategies.

Compounds	Odor Description	Batch A			Batch B			Batch C			Batch D			Batch E			Threshold	References
Higher alcohols (µg/L)																		
1-propanol	alcohol, pungent	15.67	±	1.55a	13.47	±	1.68ab	23.89	±	1.84c	10.80	±	1.08b	24.25	±	2.55c	9	[54]
1-methyl-propanol		26.97	±	0.90a	19.97	±	1.83c	19.14	±	0.81c	13.57	±	1.27b	17.42	±	1.67c		
3-methyl-1-butanol	whiskey, malt, burnt	255.87	±	33.13a	282.90	±	11.56a	138.62	±	2.95c	84.60	±	2.75b	143.70	±	2.24c	30	[54]
2-pentanol	-	22.67	±	1.94a	22.87	±	1.70a	36.77	±	3.15c	16.23	±	2.08b	35.46	±	2.41c		
1-pentanol	balsamic	11.17	±	1.46a	10.32	±	1.93a	11.80	±	1.56a	7.63	±	1.10a	12.71	±	2.62a	64	[54]
2-methyl-1-butanol	whiskey	10.13	±	0.85a	9.90	±	1.65a	4.23	±	0.51b	6.43	±	0.57b	4.35	±	1.00b	65	
1-octanol	chestnut flowers, mushroomy	0.033	±	0.00075a	0.030	±	0.00081b	0.020	±	0.00130c	0.021	±	0.00079c	0.023	±	0.00035c	0.001	[14]
tyrosol	-	58.1	±	2.79a	56.30	±	3.22a	43.5	±	2.93bc	49.43	±	4.79ac	39.3	±	4.00b		
1-heptanol	lemon, orange, copper	114.6	±	2.52a	113.77	±	6.66a	132.2	±	2.93b	111.93	±	2.54a	134.1	±	3.80b	0.2–0.3	[58]
methyl-tyo-propanol	-	85.9	±	1.72a	84.80	±	1.78a	84.1	±	2.39a	77.67	±	3.00b	84.1	±	1.90a		
2,3-butanediol	butter, creamy	382.5	±	10.58a	378.10	±	12.60a	418.5	±	17.77b	520.60	±	11.59c	434.5	±	9.76b	150	[58]
2-nonanol	fruity, green	2.2	±	0.19a	2.12	±	0.24a	2.5	±	0.21a	3.47	±	0.20b	2.5	±	0.24a	0.058	[14]
2-phenylethanol	rose, honey, spice, lilac	53.9	±	2.35a	55.33	±	2.12a	55.5	±	2.91a	87.71	±	2.14b	53.9	±	4.77a	10	[54]
methanol		27.8	±	1.40a	21.73	±	1.70a	13.4	±	1.82b	9.77	±	0.81b	14.1	±	2.11b	200	[14]
TOTAL		1067.5			1071.61			984.17			999.86			1000.28				
Esters (µg/L)																		
linalool	muscat, flowery, fruity	53.46	±	5.20a	48.50	±	4.66a	49.11	±	4.38a	124.50	±	9.81b	51.05	±	7.80a	25	[58]
4-terpineol	light aroma, wood, soil	8.47	±	1.63a	12.92	±	1.84a	10.96	±	1.81a	18.07	±	3.42b	10.30	±	0.92a	110–400	[58]
alpha-terpineol	oil, anise, mint	29.78	±	2.93a	28.43	±	3.85a	43.63	±	2.84b	44.68	±	2.25b	43.17	±	3.40b	250	[54]
geraniol	fresh, citrus, floral, green, sweet, lemon/lime	4.63	±	0.32a	4.00	±	0.26a	5.31	±	0.19a	22.42	±	3.07b	4.81	±	0.17a	15	[61]
TOTAL		96.3			93.85			109.0			209.67			109.3		3.40		
Others (µg/L)																		
hexanoic_acid	cheese, rancid, fruity	120.9	±	5.65a	124.73	±	7.07a	121.76	±	6.90a	88.94	±	5.84b	119.12	±	4.82a	670000	[60]
TOTAL		121.00			124.73			121.80			88.97			119.10				

The data are the mean of triplicates ± SD; different letters in the row denotes statistically significant differences (*p* < 0.05).
